# A Tactile Cognitive Model Based on Correlated Texture Information Entropy and Multimodal Fusion Learning

**DOI:** 10.3390/s25185786

**Published:** 2025-09-17

**Authors:** Si Chen, Chi Gao, Chen Chen, Weimin Ru, Ning Yang

**Affiliations:** 1 Research Center of Fluid Machinery Engineering and Technology, Jiangsu University, Zhenjiang 212013, China; chensi@ujs.edu.cn (S.C.); chigao@stmail.ujs.edu.cn (C.G.); gc13963146700@163.com (C.C.); ru_wm@ujs.edu.cn (W.R.); 2 School of Electrical and Information Engineering, Jiangsu University, Zhenjiang 212013, China

**Keywords:** tribology, tactile perception, texture recognition, multimodal signals, information entropy, vibroacoustics

## Abstract

(1) Background: Multimodal tactile cognition is paramount for robotic dexterity, yet its advancement is constrained by the limited realism of existing texture datasets and the difficulty of effectively fusing heterogeneous signals. This study introduces a comprehensive framework to overcome these limitations by integrating a parametrically designed dataset with a novel fusion architecture. (2) Methods: To address the challenge of limited dataset realism, we developed a universal texture dataset that leverages information entropy and Perlin noise to simulate a wide spectrum of surfaces. To tackle the difficulty of signal fusion, we designed the Multimodal Fusion Attention Transformer Network (MFT-Net). This architecture strategically combines a Convolutional Neural Network (CNN) for local feature extraction with a Transformer for capturing global dependencies, and it utilizes a Squeeze-and-Excitation attention module for adaptive cross-modal weighting. (3) Results: Evaluated on our custom-designed dataset, MFT-Net achieved a classification accuracy of 86.66%, surpassing traditional baselines by a significant margin of over 21.99%. Furthermore, an information-theoretic analysis confirmed the dataset’s efficacy by revealing a strong positive correlation between the textures’ physical information content and the model’s recognition performance. (4) Conclusions: Our work establishes a novel design-verification paradigm that directly links physical information with machine perception. This approach provides a quantifiable methodology to enhance the generalization of tactile models, paving the way for improved robotic dexterity in complex, real-world environments.

## 1. Introduction

Tactile cognition constitutes the critical physical interface for robot–environment interaction and is essential for tasks such as object recognition and dexterous manipulation, particularly in visually degraded or occluded environments [1,2,3,4], with applications ranging from industrial automation to agricultural disease monitoring where surface texture is a key indicator [5,6,7]. The human tactile system achieves efficient and robust recognition of complex textures by integrating multimodal information—including normal forces, shear forces, and high-frequency vibrations [8]. This biologically validated strategy offers a clear research avenue for enhancing the performance of robotic tactile systems. Consequently, the fusion of multimodal sensory data to improve recognition accuracy and environmental adaptability has become a central focus of research in this domain [4,9,10].

While multimodal tactile research enriches a robot’s perceptual dimensions, it also presents significant technical challenges. The notable disparities among tactile modalities in sampling frequency, signal characteristics, and temporal alignment render precise hardware synchronization and data alignment technically demanding [9,11]. Furthermore, tactile sensors are highly susceptible to ambient noise, variations in contact conditions, and inherent sensor drift. These factors often result in a low signal-to-noise ratio (SNR) in the raw data, compromising its purity and consistency and thus hindering autonomous decision-making in complex tasks. This challenge of overcoming environmental interference through the fusion of heterogeneous sensor signals is also a common theme in advanced monitoring systems [12]. Moreover, the development of robust models is hampered by limitations in existing datasets. For instance, while Devillard et al. [13] provided synchronous multimodal data, the selected textures lacked universality and failed to represent the full diversity of real-world surfaces. The tactile-visual-language dataset released by Fu et al. [14] focused more on semantic descriptions of touch rather than on fine-grained textures. The dataset from Lima et al. [15], encompassing various signals, is better suited for studying specific phenomena like anisotropic textures rather than general-purpose recognition. Similarly, though Babadian et al. [16] significantly improved object recognition by fusing tactile and visual data, their dataset did not encompass a broad range of complex textures. Given that acquiring large-scale data covering diverse contact scenarios and materials requires a substantial investment, constructing high-quality, clearly annotated, and benchmark-worthy multimodal tactile datasets remains an urgent necessity for advancing the field.

In parallel to the challenges in multimodal fusion, systems relying on a single modality also face inherent limitations. The majority of robotic tactile sensors collect unimodal signals, such as force or vibration, which precludes the comprehensive capture of rich tactile information. This fundamental constraint limits recognition performance and diminishes environmental adaptability. For example, even with advanced deep learning, the challenge of robust texture recognition persists within a single modality. As shown by Dong et al. [17], models relying exclusively on visual data are fundamentally constrained in capturing the multi-scale patterns of complex textures, highlighting the need for complementary sensory information. While Taunyazov et al. [18] improved the processing speed of unimodal signals, their method’s performance was ultimately capped by the limited information content of a single pressure modality. Likewise, the work by Chen et al. [19], despite using deep learning, was fundamentally restricted by the information capacity of vibration signals. Even innovative approaches like that of V. Zacharia et al. [20], which achieved high accuracy using a novel triboelectric sensor, are still confined to a single electrical signal modality and cannot integrate multidimensional information as human touch does. Consequently, these unimodal systems fail to emulate the integrated perceptual abilities of human touch in complex, real-world interactions.

Addressing these core challenges, this study makes two primary contributions. First, we provide a foundational data resource by constructing a benchmark texture dataset based on information entropy, designed to facilitate systematic investigations into how tactile systems perceive surface complexity. Second, we propose a multi-network fusion model for texture recognition that integrates Convolutional Neural Networks (CNNs), a channel attention mechanism (SE) [21], and Transformer modules [22]. This framework enables a systematic analysis of feature complementarity and fusion strategies, leading to a significant enhancement in the recognition of complex textures. Our work not only helps overcome current bottlenecks in robotic tactile recognition but also posits that the multimodal combination of force and acoustic signals will substantially advance the autonomous perception and intelligent interaction capabilities of robots, thereby propelling humanoid robotics toward a higher level of intelligence.

## 2. Materials and Methods

**Figure 1:**
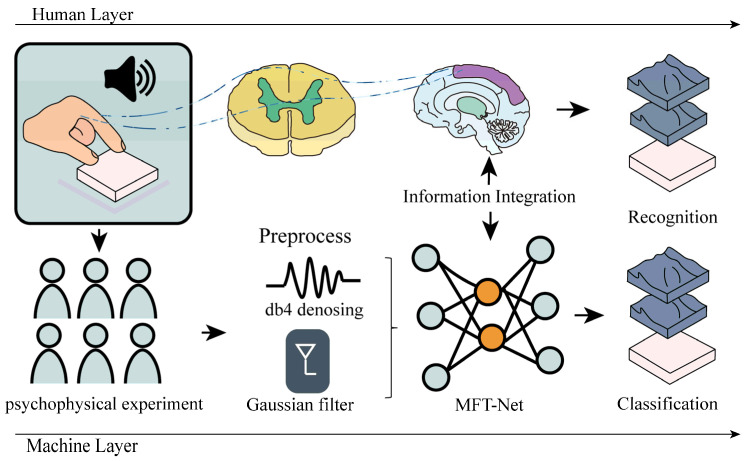
Experimental Framework for Multimodal Tactile Recognition and MFT-Net Model Architecture.

As illustrated in Figure 1, our technical pipeline begins with a set of parametrically designed texture samples. To ensure perceptual validity, these samples are first verified through psychophysical experiments. Following validation, subjects interact with the samples under a standardized data acquisition protocol, during which multimodal signals are synchronously captured. The captured raw signals are then preprocessed to remove noise and filter artifacts. Finally, these clean signals are fed into our proposed MFT-Net, which performs feature extraction, fusion, and ultimately classifies the textures.

### 2.1. Design and Fabrication of a Novel Multimodal Texture Dataset

#### 2.1.1. Texture Sample Preparation

The surfaces of the physical world exhibit a rich diversity of properties, making the creation of a dataset that exhaustively captures all real-world textures an intractable challenge. Therefore, rather than attempting to catalog this diversity, our study introduces a parametric design methodology to systematically investigate how this dimension influences tactile perception. We designed and fabricated a representative, parametric texture dataset by leveraging information theory to precisely control its information entropy. This approach offers a key advantage: by manipulating the fundamental information content of the textures, we can establish a direct and quantifiable link between a surface’s physical characteristics and a machine’s perceptual performance, a feat difficult to achieve with randomly collected samples. Our process began with generating foundational random textures using the Perlin noise algorithm [23]. We then parametrically adjusted the pixel histograms of these images via gamma transformation to construct texture samples across three distinct entropy levels. For clarity and comparison, we define the normalized entropy increment, ΔE, where ΔE = 0 represents the baseline texture, and ΔE =+1 and ΔE =−1 represent the parametrically increased and decreased entropy samples, respectively. Finally, to quantitatively validate the distinctiveness of these textures, we calculated the mutual information for all pairwise combinations according to Equation (1) and normalized the results to the [0, 1] interval.

(1) $$\;I(x,y)=\sum_{i}\sum_{j}p(x_{i},y_{j})\log_{2}\frac{p(x_{i},y_{j})}{p(x_{i})p(y_{j})}$$

**Figure 2:**
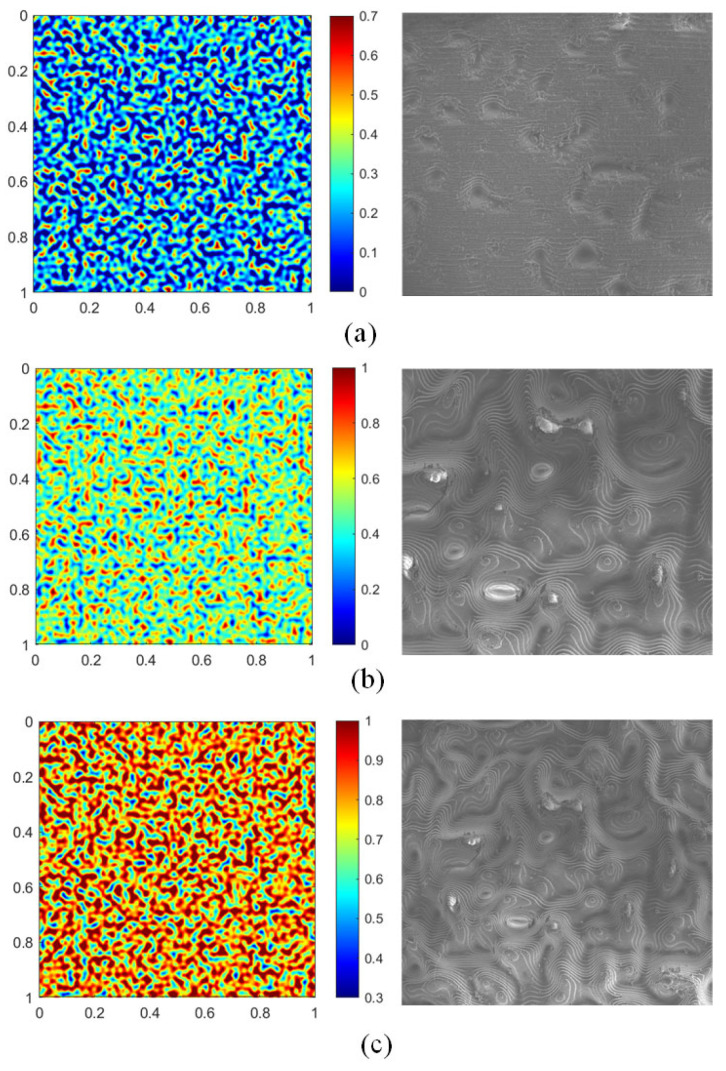
The Surface Morphologies of Information Entropy Textures: (**a**) ΔE =−1; (**b**) ΔE =0; (**c**) ΔE =1.

As detailed in Table 1 and Figure 2, our texture samples feature systematic parametric variations, making them ideal stimuli for investigating how texture characteristics influence tactile perception.

The physical samples were fabricated from their digital models using an EnvisionTEC Perfactory P4K (EnvisionTEC, Gladbeck, Germany) high-precision 3D printer. This system, which utilizes a red wax photosensitive resin and offers a build resolution of 2560 × 1600 DPI, ensured high-fidelity replication of the designed textures. Each resulting sample is a 5 cm × 5 cm square substrate with a 2.2 mm thickness, and its reverse side was precision-polished to provide a uniform contact base.

#### 2.1.2. Psychophysical Experiment

Humans perceive the physical properties of materials or textures by touching their surfaces [24]. Although the dimensions of tactile surface perception remain a subject of debate, five dimensions are currently widely recognized: macro and fine roughness, warmth/coldness, hardness/softness, and friction (encompassing wetness/dryness and stickiness/slipperiness) [24,25]. To validate the perceptual effectiveness of our texture samples, we recruited 24 healthy adult subjects (12 males, 12 females; aged 22–25 years) to perform a tactile evaluation under blindfolded conditions. They were instructed to use their right index finger to freely explore each texture and provide quantitative ratings on a 0–10 Likert scale for five perceptual dimensions. The detailed results are in Table 2.

### 2.2. Multimodal Signal Acquisition

**Figure 3:**
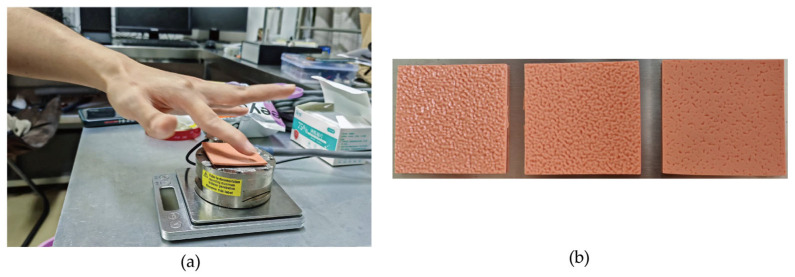
(**a**) The experimental scene for data acquisition. (**b**) The three 3D-printed physical texture samples used in the study.

Our data acquisition platform comprised an ATI Gamma six-axis force/torque sensor (ATI Industrial Automation, Apex, NC, USA), a pickup sensor, and an Audient iD4 MKII audio interface(Audient, Hampshire, UK), with a Net Box unit ensuring synchronous signal transmission to a computer for high-fidelity recording. To ensure consistent interaction, a standardized exploration protocol was established through a pilot study. Subjects were trained to maintain a normal force of 1 N, a contact angle of 30°, and a constant velocity of 10 mm/s over a 20 mm stroke. The selection of a 10 mm/s exploration speed was critical for data quality. Our pilot study revealed that this speed provides an optimal balance: it is fast enough to generate high-fidelity vibro-acoustic signals with a strong signal-to-noise ratio, yet slow enough for subjects to consistently maintain stable contact pressure, thus ensuring high repeatability across trials. This speed is also consistent with typical velocities used in prior tactile perception studies [26,27]. During each formal trial, subjects were seated ergonomically and used their right index finger to execute this controlled motion, ensuring stable contact and consistent data collection as depicted in Figure 3a. The experimental design and data acquisition parameters were meticulously controlled to ensure data quality. Force signals were captured at a 7 kHz sampling rate by the ATI sensor, while acoustic signals were recorded at 44.1 kHz by the pickup sensor. The experiment involved 20 subjects, each performing 25 trials on 3 distinct samples, with each trial lasting 12 s. To ensure high data fidelity, several control measures were implemented. The experiment was conducted in a quiet environment, and subjects rested for 3 min after every two trial sequences to mitigate sensory adaptation and fatigue. Additionally, a stringent cleaning protocol was enforced: both the subject’s finger and the sample were wiped with 75% alcohol before each trial sequence to eliminate the confounding effects of sweat contamination, thereby ensuring signal reliability.

#### Data Preprocessing

**Figure 4:**
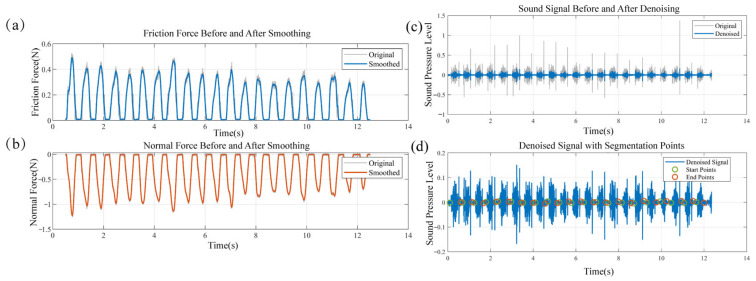
Comparison of signals before and after filtering: (**a**) Frictional force; (**b**) Normal force; (**c**) Acoustic signal; (**d**) Segmentation result of the acoustic signal.

The tactile dynamics acquisition system, equipped with an ATI Gamma multi-axis force sensor, captures three-axis orthogonal force components in real-time. The z-axis component is defined as the normal contact force, while the tangential friction force is constructed from the x–y plane components using the vector synthesis formula, $F=F_{x}^{2}+F_{y}^{2}$. To facilitate subsequent analysis and enhance the clarity of the signal’s primary trend, the raw force data underwent a smoothing procedure using a Gaussian filter with a kernel scale parameter of 500. A visual comparison of the signals before and after this smoothing process is presented in Figure 4a,b. To facilitate subsequent analysis, the signals were segmented using a sliding window (window length: 0.5 × fs; overlap: 25%), which partitioned a complete force signal into 93 segments, each representing an independent tactile perception event. Acoustic signals, being characteristically non-stationary, often have their effective information and noise intertwined in the frequency domain. We therefore employed wavelet transform for denoising, performing a 4-level signal decomposition with the Daubechies 4 (db4) wavelet basis to enable multi-scale analysis in both time and frequency. By applying a soft thresholding method to the high-frequency wavelet coefficients, random noise was effectively suppressed, and the denoised signal was obtained via wavelet reconstruction. This approach better preserves transient features compared to the traditional Fourier transform [28]. Furthermore, to address the distinct noise caused by intermittent operational interruptions from the experimenter’s reset actions, we implemented a real-time signal segmentation algorithm based on data volatility. This algorithm synergistically combines a moving average filter, a dynamic thresholding decision mechanism, and a state-tracking system to precisely extract valid signal segments. Its primary working mechanism begins with the calculation of the signal’s mean absolute slope (*AS*) to characterize its volatility.

(2) $$\;AS=\frac{1}{N-1}\sum_{i=2}^{N}|A[i]-A[i-1]|,$$

where *N* is the signal length, A[i] is the amplitude of the i-th sample point, and the standard deviation is subsequently calculated using the corresponding formula

(3) DATA_P[i]=MAE⋅DATA_P[i−1]+(1−MAE)⋅A[i],

where MAE is the smoothing factor and DATA_P represents the denoised signal. The absolute deviation between the original and smoothed signals is then calculated as

(4) DATA_A[i]=∣A[i]−DATA_P[i]∣.

Here, DATA_A reflects the local transient intensity of the signal. An adaptive threshold is subsequently generated by incorporating the signal’s statistical properties:

(5) $$\;ADD=AF1\cdot (\frac{\sigma }{AS}),$$

where σ is the signal’s standard deviation and *AF*1 is an empirical amplification factor. The term σ/AS normalizes the relationship between the volatility measure and the noise level. As the position where the action is initiated is typically close to the data peak, the detected start point is extended backward by a predefined distance, and a 50 ms buffer is appended to the end point to include any residual vibrations. Finally, the validity of each signal segment is determined using a combination of state flags and an amplitude threshold to prevent false detections. The length of all extracted valid signal segments is then uniformly normalized to 600 ms.

To address the disparity in sampling rates between the force (7 kHz) and acoustic (44.1 kHz) signals, we implemented a two-step temporal alignment process. First, for synchronization, we identified the peak in short-time energy within each touch event to serve as a common reference point. Both signal modalities were then synchronously windowed around this marker. Second, for rate matching, the high-frequency acoustic signal was passed through an anti-aliasing low-pass filter and then downsampled to 7 kHz using linear interpolation, matching the force signal’s frequency. This procedure ensures that each input pair is precisely aligned in time, eliminating hardware-induced temporal offsets. With the signals aligned, the complete dataset was prepared for model training and evaluation. The dataset was randomly partitioned into training (80%) and test (20%) sets, with corresponding label files generated for each. The meanings of these labels are detailed in Table 3**.** Finally, all data samples were normalized using min–max scaling before being fed into the model, ensuring a consistent input range for optimal network performance.

### 2.3. The Proposed Tactile Cognitive Model

**Figure 5:**
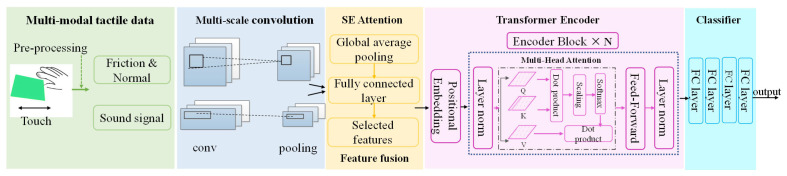
The architecture of the proposed MFT-Net.

To address the concurrent presence of local transient features and global temporal dependencies within multimodal tactile signals, we propose a novel end-to-end deep learning model, designated as MFT-Net. While end-to-end Transformer-based models like DETR have shown promise in various detection tasks, their direct application can be challenging due to computational complexity and slow convergence, necessitating architectural innovations for specific domains [29]. The core concept of this model is to synergistically leverage the robust local feature extraction capabilities of Convolutional Neural Networks (CNNs) [30,31], the cross-modal feature weighting capacity of Squeeze-and-Excitation (SE) networks, and the global temporal modeling power of the Transformer architecture. The overall framework of the model is depicted in Figure 5.

To achieve multiscale feature extraction, we designed a parallel dual-branch CNN module [32,33] to process the aligned force and tribo-acoustic signals independently. Each branch comprises three layers of 1D depthwise separable convolutions [33,34]. By cascading three convolutional kernels of varying scales, this architecture progressively captures short-term fluctuations, medium-range correlations, and long-range trend features from the time-domain signals. As the force-signal branch processes two distinct signal types (tangential and normal forces), each convolutional layer in this branch also incorporates a spatial convolution to concurrently extract features from both. Each convolutional layer is followed by a Batch Normalization layer and a ReLU activation function to mitigate the vanishing gradient problem and accelerate convergence. To reduce feature dimensionality and redundancy, an adaptive average pooling layer is deployed after the third convolutional layer, compressing the feature sequence length to 200. This step retains critical pattern information while eliminating redundant data. Subsequently, the force and acoustic feature tensors output by the dual branches are concatenated along the channel dimension and fed into a SE attention mechanism for cross-modal interaction and feature selection [35]. The SE block generates a channel-wise statistic vector via global average pooling. This vector is then passed through two fully connected layers to learn the weight coefficients for each channel, thereby enabling dynamic recalibration of the feature channels. This process selectively emphasizes the most informative features from both the force and acoustic signals. The squeeze operation, $F_{sq}\left(\cdot \right)$, applies global average pooling to the feature map U to produce a 1×1×C vector, where C is the number of channels. The output of this operation, $z_{c}$, is given by (6) The subsequent excitation operation, $F_{ex}\left(\cdot \right)$ processes $z_{c}$ through two fully connected layers, $W_{1}$ and $W_{2}$, to obtain the final channel weights, $s_{c}$:

(6) $$\;z_{c}=F_{sq}\left(u_{c}\right)=\frac{1}{H\times W}\sum_{i=1}^{H}\sum_{j=1}^{W}u_{c}\left(i,j\right),$$

(7) $$s_{c}=F_{ex}\left(z_{c},W\right)=\sigma \left(W_{2}\delta \left(W_{1}z_{c}\right)\right).$$

The optimized feature map from the SE block is then input into a Transformer encoder to model global temporal dependencies using its multi-head self-attention mechanism. The encoder consists of 6 stacked layers, with each layer containing 10 parallel attention heads. This mechanism computes the association weights between different temporal positions through Query-Key-Value mappings. The matrices Q, K, V are obtained via linear transformations of the input vector x, and $d_{k}$ is the dimension of the key vectors. The attention is calculated as follows:

(8) $$\;\mathrm{Attention}(Q,K,V)=\mathrm{Softmax}(\frac{QK^{T}}{\sqrt{d_{k}}})V,$$

(9) $$head_{i}=\mathrm{Attention}(Q{W_{i}}^{Q},K{W_{i}}^{K},V{W_{i}}^{V}),$$

(10) $$\mathrm{MultiHead}\left(Q,K,V\right)=\mathrm{Concat}\left(head_{1},\ldots ,head_{h}\right)W^{O}.$$

This mechanism enables the model to capture long-range causal relationships within the tactile signals, thereby overcoming the limited receptive field inherent in traditional CNNs. By analyzing the global correlations between different temporal positions in the feature sequence, long-term temporal features are further extracted. Concurrently, positional encoding and residual connections are incorporated to ensure the faithful propagation of sequential information. Finally, the high-order features output by the Transformer are fed into a four-layer fully connected network. Dropout is employed to mitigate overfitting, and the network ultimately outputs the probability distribution for each class. The structural parameters of the model are detailed in Table 4.

### 2.4. Experimental Setup and Fusion Strategies

The model was trained and evaluated within the PyTorch framework (version 1.12). The network computes the predicted output via forward propagation, and the cross-entropy loss function is employed to quantify the discrepancy between the predictions and the ground-truth labels. Weights are updated using the backpropagation algorithm, with the Adam optimizer selected to implement the parameter updates. The hyperparameters for the Adam optimizer were set to β1 = 0.5 and β2 = 0.99. A batch size of 72 was used, and the initial learning rate was set to 1 × 10^−4^, coupled with a linear warmup strategy to mitigate gradient oscillations in the early stages of training. To prevent overfitting, both Dropout and L2 weight decay were incorporated into the model. The Dropout rate was set to 0.4, and the L2 weight decay was configured to 1 × 10^−4^. A complete summary of these training parameters is provided in Table 5.

In multimodal signal classification tasks, the effectiveness of inter-modal information fusion directly dictates the model’s capability to represent complex operational conditions. To leverage the complementary characteristics of force and acoustic signals, we designed a synergistic mechanism that combines data-level alignment with feature-level interaction, thereby achieving efficient force-acoustic collaborative perception. Data-level alignment, as implemented in the preprocessing stage, ensures that all force-acoustic signal pairs input to the network are strictly synchronized in the time domain through event localization and signal resampling. Feature-level fusion can be categorized into three main strategies: early, intermediate, and late fusion. Early fusion involves concatenating the raw data of the force and acoustic modalities at the input layer or in shallow network layers (e.g., stacking multi-channel signals), effectively representing fusion at the data or low-level feature level. Late fusion, also known as decision-level fusion, entails training independent classification models for each modality and then combining their outputs at the final layer, typically through weighted voting or probability averaging. Within the MFT-Net framework, we implement an intermediate fusion strategy where feature fusion is accomplished via the adaptive cross-modal feature weighting of SE attention mechanism. Specifically, the outputs from the force and vibration signal branches are concatenated along the channel dimension to form a joint feature representation. The SE module then generates a channel-wise statistic vector by applying global average pooling to each channel. A two-layer fully connected network learns the corresponding channel weights from this vector. These learned weights are then multiplied channel-wise with the original feature map, enabling an adaptive selection of cross-modal features. The channel weights learned by the SE module’s fully connected layers implicitly contain an assessment of modal quality. For instance, if the vibration signal is contaminated by ambient noise, the SE network will automatically down-weight the activation of its corresponding channels, and vice versa. A similar adaptive weighting is applied to the force signal channels, ensuring a robust fusion process.

### 2.5. Baseline Models, Feature Selection, and Performance Evaluation Metrics

To evaluate the performance of MFT-Net, we benchmarked it against two traditional machine learning models on our custom texture dataset. These baseline models, specifically a Random Forest [36,37] and a K-Nearest Neighbors [38] classifier, relied on a comprehensive set of handcrafted features. As detailed in Table 6, this feature set encompassed statistical, time-domain, and frequency-domain characteristics, providing a robust point of comparison for our end-to-end approach.

All models were evaluated on the identical training and test sets, strictly adhering to an 8:2 data partitioning principle for training and testing, respectively. To assess the recognition accuracy, we calculated several performance metrics, including *Acc*, Precision, Recall, $F_{1}$*-score*, Balanced Accuracy (BAC), and the Kappa coefficient (κ). These metrics are defined by the following equations:

(11) $$\;\mathrm{Acc}=\frac{TP}{total},$$

(12) $$\mathrm{Precision}=\frac{TP}{TP+FP},$$

$$\mathrm{Recall}=\frac{TP}{TP+FN},$$

(13) $$F_{1}=\frac{2\mathrm{Precision}\cdot \mathrm{Recall}}{\mathrm{Precision}+\mathrm{Recall}},$$

(14) $$\mathrm{BAC}=\frac{\mathrm{Recall}+\mathrm{Specificity}}{2}=\frac{1}{2}(\frac{TP}{TP+FN}+\frac{TN}{TN+FP}),$$

(15) $$\kappa =\frac{p_{o}-p_{e}}{1-p_{e}},$$

(16) $$p_{o}=\frac{TP+TN}{TP+TN+FP+FN},$$

(17) $$p_{e}=\frac{\left(TP+FP)(TP+FN)+(TN+FN)(TN+FP\right)}{(TP+TN+FP+FN{)}^{2}}$$

## 3. Results and Discussion

### 3.1. Psychophysical Experiment Results

**Figure 6:**
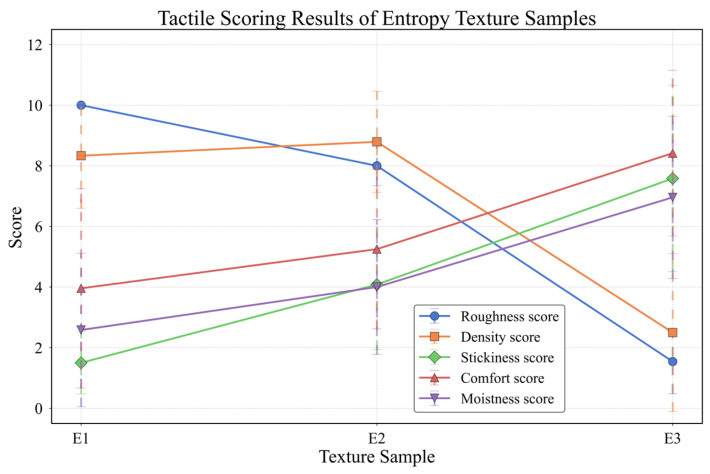
Tactile scoring results of texture samples.

The psychophysical rating results, shown in Figure 6, reveal clear perceptual trends directly correlated with the textures’ information entropy. A strong inverse relationship was observed for macro-roughness: as entropy decreased from E1 to E3, the surfaces became physically smoother, and subjects assigned progressively lower roughness scores. Conversely, ratings for stickiness, comfort, and perceived wetness showed a positive correlation, increasing steadily as the surfaces became smoother. Notably, the Entropy_3 sample, having the lowest entropy, was perceived as the least rough, most comfortable, stickiest, and moistest, confirming the consistent trend across these perceptual dimensions. Interestingly, the fine-roughness scores for E1 and E2 were comparable, likely because while E1 has a higher density of sharp protrusions, their underlying texture distributions are similar, resulting in a consistent fine-scale tactile sensation.

### 3.2. Performance Evaluation of the MFT-Net Model

As shown in Table 7, MFT-Net achieved a classification accuracy of 86.66%, outperforming the best-performing traditional baseline by a significant margin of 21.99%. This substantial performance gap highlights the fundamental limitations of relying on handcrafted features, which struggle to fully capture the complex, high-dimensional, and non-linear patterns inherent in tactile signals. In contrast, MFT-Net’s end-to-end architecture excels precisely because it automatically learns more discriminative, hierarchical features directly from the raw data. This principle, where deep learning models extract features directly from rich, pixel-level or raw sensor data, has been shown to outperform traditional feature engineering approaches in other domains [39,40]. This result not only confirms the superior capability of our model but also validates the necessity of deep learning frameworks for tackling such complex texture recognition tasks.

### 3.3. Validation on a Public Tactile Dataset

To address the generalizability of our proposed MFT-Net, we extended our evaluation to a publicly available real-world tactile dataset: the TUM Tactile Texture Database [41]. This benchmark dataset contains multimodal tactile signals collected from various textures, providing a challenging testbed for model validation. We benchmarked our MFT-Net against several state-of-the-art methods, with the comparative results summarized in Table 8. Our model’s architecture offers a more effective way to handle sequential tactile data compared to other models, the Transformer component in MFT-Net is better at capturing long-range dependencies and complex temporal relationships within the signals. This allows for a more holistic understanding of the entire tactile interaction, resulting in higher classification accuracy.

### 3.4. Ablation Study

To systematically dissect the sources of MFT-Net’s superior performance, we conducted a series of ablation studies designed to investigate three critical aspects: the necessity of multimodal fusion, the individual contribution of each core architectural component, and the comparative effectiveness of our chosen fusion strategy against common alternatives.

#### 3.4.1. Ablation Analysis of Modalities and Model Components

To validate the contributions of both its multimodal inputs and core architectural components, we conducted a series of ablation studies on MFT-Net.

**Figure 7:**
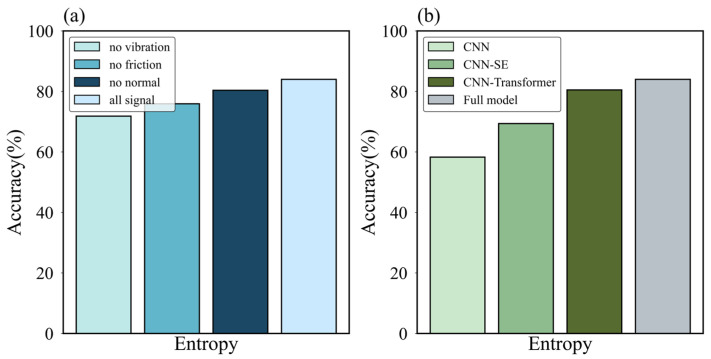
(**a**) Results of the ablation experiments of the model components. (**b**) Results of the ablation experiments on tactile signals.

First, we evaluated the importance of each sensory modality through modal-dropout experiments. As shown in Figure 7a, removing any single modality degraded performance, confirming their complementarity. The acoustic signal proved most critical; its removal caused the accuracy to drop to 71.84%. The friction force was the second most influential factor. The normal force, while having the least impact, still contributed positively to the model’s performance, as its removal led to a decrease in accuracy. This outcome highlights that the acoustic signal likely captures the surface’s micro-geometry, while the force signals reflect its macroscopic frictional properties, making their fusion essential for high-accuracy recognition.

Next, we assessed the efficacy of each core architectural component. The results, shown in Figure 7b, revealed that the Transformer module was the most significant contributor; its removal caused a substantial 14.63% drop in accuracy. The SE attention block also proved crucial, with its absence leading to a 4% performance decrease. These findings validate our design choices: the Transformer’s impact underscores the importance of modeling long-range temporal dependencies, while the SE block’s contribution demonstrates the value of adaptively weighting cross-modal features, a more intelligent approach than simple concatenation. Interestingly, while data-driven attention mechanisms are powerful, some studies suggest that integrating features derived from physical properties, such as texture analysis via GLCM, can offer more consistent performance improvements in certain classification tasks compared to attention mechanisms alone [46]. This highlights the potential synergy between abstract feature learning and physics-informed feature extraction in complex perception problems.

Ultimately, these ablation studies confirm that MFT-Net’s powerful performance stems from a carefully designed synergy: the CNNs capture local details, the Transformer models global context, and the SE block intelligently fuses them.

#### 3.4.2. Analysis of Fusion Strategy Effectiveness

Our performance comparison, presented in Table 9, validates the superiority of our SE-based intermediate fusion strategy, which achieved an accuracy of 81.04%. Its success stems from its ability to effectively balance the unique characteristics of each modality before integration. Unlike alternative strategies, our approach first allows each modality-specific CNN branch to independently learn rich, high-level feature representations. Only then does the SE attention block act as an “intelligent gateway,” dynamically assessing the relevance of these features and weighting them for optimal fusion.

This method effectively avoids the pitfalls of the alternatives. Early fusion, by contrast, struggles because it merges the physically distinct force and acoustic signals at a raw or low-level stage, leading to inefficient feature learning. Late fusion fails for the opposite reason: by keeping the modalities separate until the final decision layer [47,48], it prevents the model from exploiting the rich, complementary information present in the intermediate feature layers. Our intermediate fusion strategy thus strikes a critical balance, enabling effective cross-modal interaction without premature or delayed integration.

### 3.5. Cross-Validation of Information-Theoretic Parameters and Model Performance

**Figure 8:**
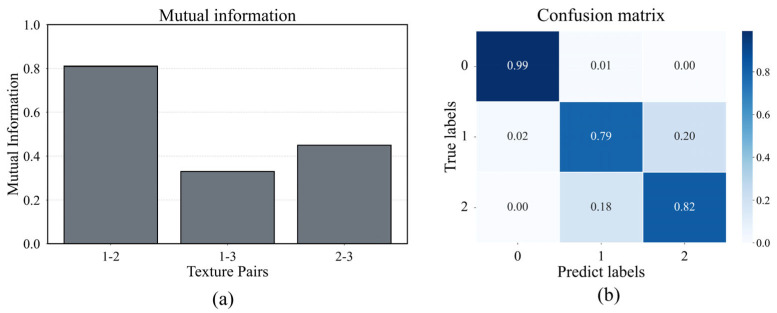
(**a**) Normalized mutual information between textures in the two datasets. (**b**) Confusion matrix for the Entropy dataset.

Our analysis reveals a direct correlation between the information-theoretic properties of the textures and our model’s classification performance. As shown in Figure 8, the normalized mutual information (NMI) [49] quantifies the informational similarity between textures. For instance, the high NMI between E1 and E2 (0.81) indicates significant shared information, predicting they would be difficult to distinguish. Conversely, the low NMI between E1 and E3 (0.33) signals high discriminability.

This theoretical prediction is precisely mirrored in the model’s confusion matrix. The highest rates of misclassification consistently occurred between samples with high NMI, while our MFT-Net model achieved zero mutual misclassifications between E1 and E3, the pair with the lowest NMI. This strong correspondence serves two key purposes: first, it empirically validates our dataset’s design by demonstrating that its information-theoretic structure is perceptually and computationally relevant. Second, it establishes a quantifiable link between the physical information content of a surface and the cognitive challenge it presents to a machine perception system, substantiating the strong correlation between information content and tactile cognition.

### 3.6. Limitation

This study establishes a systematic research paradigm for tactile perception by linking parametric sample design with algorithmic recognition and information-theoretic analysis. By demonstrating a quantifiable correlation between a texture’s information content and a model’s recognition performance, we offer a novel methodology for designing and validating multimodal tactile systems. Our MFT-Net model serves as an effective framework for this paradigm, proving valuable for applications that demand fine-grained surface perception.

However, we acknowledge several limitations. First, our investigation was confined to a single material and did not incorporate other physical dimensions such as thermal properties. Second, the tactile exploration was restricted to a fixed, controlled strategy, which differs from the dynamic interactions common in natural environments. Consequently, the model’s generalization to a broader range of materials and dynamic conditions requires further investigation.

Immediate future work will address these limitations. The dataset can be expanded by applying our parametric design methodology to new materials and by integrating additional modalities, such as temperature. Furthermore, we plan to investigate the model’s robustness under unconstrained, dynamic exploratory conditions to enhance its real-world applicability.

Looking further ahead, our framework is poised to advance multimodal tactile rendering. By leveraging the established link between physical parameters and perceptual signals, it becomes possible to generate realistic virtual force and vibro-acoustic feedback directly from a texture’s information properties (e.g., entropy value), significantly enhancing immersion in virtual reality and teleoperation. Finally, this paradigm opens avenues for cross-modal generation, such as synthesizing the tactile signals produced by touching a surface from its visual image alone. Success in this area would not only enrich the interactive dimensions of virtual content but also provide a powerful tool for investigating the complex perceptual transformations between vision and touch.

## 4. Conclusions

This study introduces a comprehensive “design-validation-recognition” research paradigm. This paradigm integrates the design of canonical textures using information theory, their validation through psychophysical experiments to ground them in human perception, and their classification using a novel multimodal fusion model.

Experimental results demonstrate the success of this paradigm: our MFT-Net model achieved a classification accuracy of 86.66%, significantly outperforming traditional methods. More importantly, our analysis reveals a strong correlation between the textures’ mutual information and the model’s classification confusion. This linkage provides a new information-theoretic basis for predicting and understanding the performance limits of tactile systems, moving beyond simple accuracy metrics.

In conclusion, this research provides more than just an effective recognition model; it establishes a quantifiable bridge between the physical information content of a surface and the cognitive challenge it presents to a robot. This approach offers a new pathway for developing more sophisticated autonomous perception, paving the way for robots that can interact with the world with greater intelligence and dexterity.

## Figures and Tables

**Table 1:** Surface Parameters of Information Entropy Textures.

Sample ID	Entropy	Normalized Mutual Information
Entropy1	7.5	1–2: 0.81
Entropy2	6.5	1–3: 0.33
Entropy3	5.5	2–3: 0.45

**Table 2:** Semantic Descriptions of the Dimensions of Tactile Perception.

Category	Dimension	Description
Psychophysical	Macro-roughness	Uneven, Uniformly flat, Embossed sensation
Psychophysical	Fine roughness	Coarse, Fine, Sparse/Dense
Psychophysical	Stickiness/Slipperiness	Low grip, Slippery, High adhesion sensation
Psychophysical	Wetness/Dryness	Moist, Dry
Affective	Comfort Level	Pleasant and comfortable to the touch

**Table 3:** Meanings of the Labels in the Dataset.

Dataset	0	1	2
Entropy	E1 = 5.5	E2 = 6.5	E3 = 7.5

**Table 4:** Setting of Important Parameters of the Model.

Layer	Kernel/Key
Conv1	1 × 60
Conv2	1 × 40
Conv3	1 × 20
Conv4,5,6	2 × 1
AvgPooling	1 × 200
Query/Key/Value	80
Depth	6
Num_hidden	80
Head	10

**Table 5:** Setting of Training Parameters of the Model.

Parameter	Batch Size	Learning Rate	Epoch	Optimizer	L2	Dropout
Key	72	0.0001	200	Adam	0.0001	0.4

**Table 6:** Summary of Tactile Signal Feature Extraction.

Force Signal Features	Vibration Signal Features
Mean, Variance	Spectral Centroid, Spectral Entropy
Root Mean Square (RMS)	Power Spectral Density (PSD), Zero-Crossing Rate (ZCR)
Friction Coefficient	Short-Time Energy
Energy	Mel-Frequency Cepstral Coefficients (MFCCs)
Spectral Centroid, Bandwidth	Grayscale Histogram: Mean, Variance, Entropy
Spectral Entropy	Gray-Level Co-occurrence Matrix (GLCM): Energy, Entropy, Inertia
Power Spectral Density (PSD)	Fractal Dimension
Skewness	Skewness
Kurtosis	Kurtosis

**Table 7:** Performance Comparison of MFT-Net with Traditional Baseline Methods.

Model	Acc	Precision	Recall	F1	BAC	Kappa
MFT-Net	86.66%	84.53%	85.03%	83.15%	84.13%	83.74%
RF	63.33%	62.44%	63.02%	60.75%	61.43%	61.83%
KNN	64.67%	62.51%	61.33%	61.89%	63.59%	60.94%

**Table 8:** Performance comparison on the TUM Tactile Texture Database.

Model	Data	Accuracy	Precision	Recall	F1 Score
WCMAL [42]	acceleration, image	88.6%	86.5%	84.8%	85.6%
HapticNet [43]	acceleration, image	91%	89.5%	87.3%	88.4%
Handcrafted multimodal features [41]	vibration, acceleration, friction, image	75%	72.6%	70%	71.3%
Handcrafted multimodal features [44]	vibration, acceleration, friction, image	90.5%	89.1%	87.3%	88.2%
CNN-LSTM [45]	vibration, acceleration, friction	91.7%	89.3%	88.9%	90.1%
Proposed Multi-Model Fusion Network	vibration, acceleration, friction	93.2%	91.7%	90.5%	89.3%

**Table 9:** Comparison of the effects of multi-modal fusion strategies.

Fusion Strategy	Early Fusion	Intermediate Fusion (SE)	Late Fusion
Entropy	71.82%	81.04%	75.91%

## Data Availability

Dataset available on request from the authors.
